# Transscleral vs endoscopic cyclophotocoagulation: safety and efficacy when combined with phacoemulsification

**DOI:** 10.1186/s12886-023-02877-6

**Published:** 2023-03-30

**Authors:** Abraham Nirappel, Emma Klug, Cameron Neeson, Mari Chachanidze, Hani El Helwe, Nathan Hall, Ta C. Chang, Lucy Q. Shen, David Solá-Del Valle

**Affiliations:** 1 Massachusetts Eye and Ear, Department of Ophthalmology, Harvard Medical School, 243 Charles St, Boston, MA USA; 2 Bascom Palmer Eye Institute, University of Miami Leonard M. Miller School of Medicine, Miami, FL USA

**Keywords:** Microinvasive glaucoma procedure, MicroPulse, Endoscopic cyclophotocoagulation, Survival-success rate

## Abstract

**Purpose:**

To compare the effectiveness and safety of phacoemulsification combined with endoscopic cyclophotocoagulation (phaco/ECP), phacoemulsification combined with MicroPulse transscleral cyclophotocoagulation (phaco/MP-TSCPC), and phacoemulsification alone (phaco) in the treatment of coexisting cataract and glaucoma.

**Methods:**

Retrospective cohort study of consecutive cases at Massachusetts Eye & Ear. The main outcome measures were the cumulative probabilities of failure between the phaco/ECP group, phaco/MP-TSCPC group, and the phaco alone group with failure defined as reaching NLP vision at any point postoperatively, undergoing additional glaucoma surgery, or the inability to maintain ≥ 20% IOP reduction from baseline with IOP between 5–18 mmHg while maintaining ≤ baseline medications. Additional outcome measures included changes in average IOP, number of glaucoma medications, and complication rates.

**Results:**

Sixty-four eyes from 64 patients (25 phaco/ECP, 20 phaco/MPTSCPC, 19 phaco alone) were included in this study. The groups did not differ in age (mean 71.04 ± 6.7 years) or length of follow-up time. Baseline IOPs were significantly different between groups (15.78 ± 4.7 mmHg phaco/ECP, 18.37 ± 4.6 mmHg phaco/MP-TSCPC, 14.30 ± 4.2 mmHg phaco alone, *p* = 0.02). Primary open-angle glaucoma was the most common type of glaucoma in the phaco alone (42%) and phaco/ECP (48%) groups while mixed-mechanism glaucoma was the most common type in the phaco/MP-TSCPC group (40%). Surgical failure was less likely in eyes in the phaco/MP-TSCPC (3.40 times, *p* = 0.005) and phaco/ECP (1.40 times, *p* = 0.044) groups compared to phaco alone based on the Kaplan–Meier survival criteria. These differences maintained statistical significance when differences in preoperative IOP were taken into account using the Cox PH model (*p* = 0.011 and *p* = 0.004, respectively). Additionally, surgical failure was 1.98 times less likely following phaco/MP-TSCPC compared to phaco/ECP (*p* = 0.038). This difference only approached significance once differences in preoperative IOP were accounted for (*p* = 0.052). There was no significant difference in IOP reduction at 1 year between groups. Mean IOP reductions at 1 year were 3.07 ± 5.3 mmHg from a baseline of 15.78 ± 4.7 in the phaco/ECP group, 6.0 ± 4.3 mmHg from a baseline of 18.37 ± 4.6 in the phaco/MP-TSCPC group and 1.0 ± 1.6 from a baseline of 14.30 ± 4.2 mmHg in the phaco alone group. There were no differences in complication rates among the three groups.

**Conclusions:**

Both Phaco/MP-TSCPC and phaco/ECP appear to provide superior efficacy for IOP control when compared to phaco alone. All three procedures had similar safety profiles.

**Supplementary Information:**

The online version contains supplementary material available at 10.1186/s12886-023-02877-6.

## Introduction

Laser cyclophotocoagulation has been proposed as a potentially safe and effective intraocular pressure (IOP)-lowering tool. During cyclophotocoagulation, the laser can be delivered through either a transscleral or an endoscopic approach to the ciliary processes in an effort to decrease aqueous production [1, 2]. In the endoscopic method, the surgeon directly visualizes and targets the ciliary processes using a video camera, while the transscleral method involves transmitting a beam of laser energy to the ciliary body through the overlying sclera. When combined with phacoemulsification, laser cyclophotocoagulation can potentially decrease IOP without increasing the surgical risks. Theoretically, the endoscopic approach may allow for more precise targeting and thus limit collateral tissue damage when compared to the transscleral approach, although the longer operative time and greater intraocular manipulation may offset these potential advantages. Furthermore, whereas the transscleral approach can deliver laser circumferentially during one treatment session, the endoscopic approach can only treat up to 270 degrees of ciliary processes per session without creating an additional corneal incision. Currently, there are no high-quality studies comparing the two approaches of cyclophotocoagulation with a phacoemulsification-only arm as a control, and the relative efficacy and safety of these two approaches remain unknown.

In this study, we compared the outcomes of combined phacoemulsification and MicroPulse transscleral cyclophotocoagulation (phaco/MP-TSCPC) and combined phacoemulsification and endoscopic cyclophotocoagulation (phaco/ECP) to phacoemulsification alone. Given the popularity of various microinvasive glaucoma surgery (MIGS) combined with cataract surgery in glaucoma patients, comparing the efficacy of these combined procedures is a topic of significant clinical interest.

## Methods

### Study design

The study protocol was approved by the Institutional Review Board of Massachusetts Eye and Ear (MEE), and the data collection methods abided by the Declaration of Helsinki and the Health Portability and Accountability Act. We identified consecutive patients who had undergone either phaco/MP-TSCPC, phaco/ECP, or phaco alone at MEE from March 2011 to December 2019 using financial claims data. Patients were included for analysis if: 1) they had undergone clear-corneal phacoemulsification, 2) either no other combined procedure, ECP, or MP-TSCPC, 3) had a glaucoma diagnosis at the time of the index procedure, and 4) had a follow up of at least 6 weeks.

Patients were excluded if they were under the age of 18 years at the time of the procedure, had history of prior phacoemulsification, ECP, or phaco/MP-TSCPC, received less than 300° of ECP, or if they had juvenile open-angle glaucoma (JOAG). If both eyes were treated with the same combination of procedures, only one eye per patient was randomly selected using a random number generator.

Demographic and ophthalmic data were collected from the preoperative visit. Preoperative IOP was calculated as an average of the IOP readings from the two visits immediately preceding treatment. Glaucoma severity was determined as mild, moderate or severe based on the glaucoma staging codes determined by the American Glaucoma Society, or as indeterminate if automated visual field data was not available [3]. Fixed-dose combination glaucoma medications were counted by the number of their constituent agents. Intraoperative data collected included laser power and duration of treatment.

Postoperative data on IOP, number of glaucoma medications, visual acuity, subsequent IOP-lowering procedures, and the presence of complications were recorded. Complications collected throughout the follow-up period are defined as postoperative findings of hypotony (defined as IOP ≤ 5 mmHg), cystoid macular edema (CME), and inflammation evidenced by the presence of any anterior chamber cells. Surgical failure criteria are defined below.

### Surgical procedure

#### Phaco/MP-TSCPC

A peribulbar block was performed by anesthesia with 5 mL of 1% preservative-free lidocaine and 0.375% preservative-free bupivacaine, along with monitored anesthesia care. The patient’s operative eye and ocular adnexa were then sterilized with 5% Betadine solution and draped in the usual sterile ophthalmic fashion. A sterile lid speculum was placed in the operative eye. We then proceeded with standard phacoemulsification [4]. Afterwards, two 90-s applications (one in the inferior quadrant and one in the superior quadrant) of MP-TSCPC were done with a power between 2000–2400 mW at a 31.3% duty cycle using the Generation 1 probe.

Power was titrated based on the need for IOP lowering at the discretion of the surgeon. The 3 and 9 o’clock meridians were avoided. The main wound and the paracentesis wounds were then hydrated and found to be free of any leaks. All patients received an intracameral injection of antibiotics. Unless patients had medical comorbidities (e.g., uncontrolled diabetes or hypertension) that prevented the use of systemic steroids, patients received 1 g of IV methylprednisolone intraoperatively followed by a 6-day PO methylprednisolone taper (Medrol dose pack). IOP-lowering medications were given on postoperative day 1 at the discretion of the attending surgeon.

#### Phaco/ECP

A peribulbar block was performed by anesthesia with 5 mL of 1% preservative-free lidocaine and 0.375% preservative-free bupivacaine, along with monitored anesthesia care. The patient’s operative eye and ocular adnexa were then sterilized with 5% Betadine solution and draped in the usual sterile ophthalmic fashion. A sterile lid speculum was placed in the operative eye. Following standard phacoemulsification, endocyclophotocoagulation was applied to the ciliary processes through the main wound with a power between 0.14 W and 0.40 W, which was titrated to ciliary body shrinkage. An additional wound was created with a keratome to complete the treatment for a total of 300° to 360°. OVD material was removed with irrigation and aspiration. The corneal incisions were watertight at the end of the procedure. Triamcinolone was injected into the anterior chamber of eye and patients were instructed to resume glaucoma eye drops on the evening following surgery at the surgeon’s discretion.

### Outcome measures

The primary outcome measures included the average reduction in IOP and Kaplan–Meier (KM) survival. Two separate failure criteria were defined.

Further glaucoma surgery was included as a failure criterion. We have edited the 20% IOP reduction criteria to the following to make it clearer.

Twenty percent IOP reduction criteria: Failure was defined as the inability to maintain ≥ 20% IOP reduction from baseline with IOP between 5–18 mmHg while having a reduction in glaucoma medication burden for two consecutive follow up visits. The latter follow-up visit was used as the failure date. Patients who encountered a disqualifying event including procedures that may impact IOP, such as a secondary glaucoma surgery, or ocular laser were considered as failures on the date of the additional procedure.

Goal IOP criteria: Failure was defined as the inability to attain a preoperatively designated goal IOP (determined to be either ≥ 30% reduction from where the glaucoma specialist noted progression or the IOP at which the glaucoma specialist thought that the patient should be to prevent further progression based on their presentation), or if patients were at goal IOP preoperatively on glaucoma medications, failure to maintain goal IOP while reducing glaucoma medication burden on two consecutive follow-up visits after 30 days OR need for additional glaucoma surgery.

Additional outcome measures including changes in IOP, number of glaucoma medications, visual acuity, and the prevalence of postoperative complications continued to be recorded after the failure date used in the KM curves. The survival analysis follow-up time may thus differ from the general follow-up time included in other areas of the paper (e.g., Tables).

### Statistical analysis

One-way ANOVA tests were conducted to determine if there were significant differences between the three groups in terms of average IOP reduction or medication burden reduction. Tukey’s honest significant difference (Tukey HSD) post-hoc tests adjusted for multiple comparisons were conducted to make pairwise comparisons between each of the three groups. One-way ANOVA and Tukey HSD post-hoc tests were also used to detect if there were significant differences between the three groups in terms of preoperative IOP or number of glaucoma medications. Chi-squared tests were also used to determine if the two groups differed significantly in terms of their preoperative characteristics or in the prevalence of postoperative complications.

KM survival curves were constructed to compare the long-term cumulative probabilities of success between the groups that received either phaco alone, phaco/ECP, or phaco/MP-TSCPC. Pairwise log-rank tests with adjustments for multiple comparisons were used to determine if the survival curves of the three groups differed significantly from one another. Cox proportional-hazard (Cox PH) regression analyses were fit to determine the effects of age, sex, preoperative IOP, type of glaucoma, and preoperative medication burden on the hazard of failure. A life table was created to compare the cumulative probabilities of survival at various selected time points.

## Results

### Demographics

**Fig. 1:**
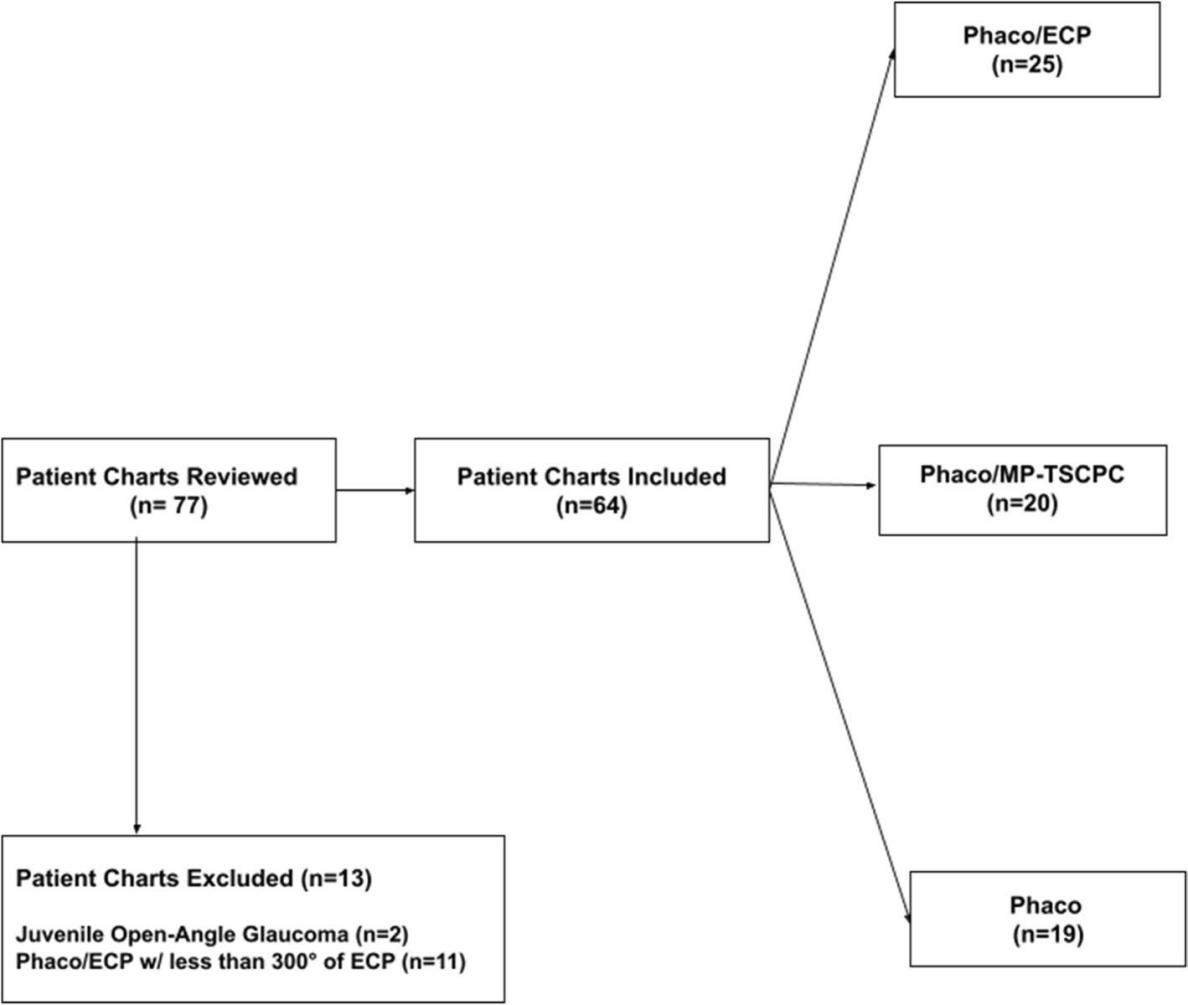
Flowchart depicting exclusion criteria and sample sizes for each arm

A total of 64 eyes from 64 patients were included in this study; 25 in the phaco/ECP group, 20 in the phaco/MPTSCPC group, and 19 in the phaco alone group (Fig. 1). Total follow-up time ranged from 41 to 508 days. The mean follow-up time for the survival analysis was 215.27 ± 34.36 days in the phaco/ECP group, 256.39 ± 42.33 days in the phaco/MP-TSCPC group, and 112.82 ± 7.87 in the phaco alone group (*p* = 0.021). The median follow-up times for the survival analysis were 201 (IQR: 35) in the phaco/ECP group, 243 (IQR: 46) in the phaco/MP-TSCPC group and 88 (IQR: 27) in the phaco alone group. The mean follow-up time for which data on IOP, medication, visual acuity and complications were captured was 356.37 in the phaco/ECP group, 384.50 in the phaco/MP-TSCPC group, and 324.66 in the phaco alone group (*p* = 0.34). Preoperatively, no significant differences were found among the 3 groups in gender (overall, 55.5% female, *p* = 0.06), visual acuity (overall average ± standard deviation [SD], 0.59 ± 0.35, logarithm of the minimum angle of resolution, *p* = 0.07), and age 71.0 ± 10.2 years (*p* = 0.34). No patient reached NLP vision at any point in the study. Mean baseline IOP was significantly higher in the phaco/MP-TSCPC group than in the phaco alone group (18.37 ± 4.6 mmHg vs 14.30 ± 4.2 mmHg, *p* = 0.02). Primary open-angle glaucoma (POAG) was the most common type of glaucoma in the phaco alone (42%) and phaco/ECP (48%) groups while mixed-mechanism glaucoma was the most common type in the phaco/MP-TSCPC group (40%). Both the phaco/MP-TSCPC (63%, *p* < 0.01) and the phaco/ECP (32%, *p* = 0.03) groups had significantly lower proportions of severe glaucoma patients than the phaco alone groups (92%) (Table 1).

**Table 1:** Baseline Characteristics

	**Baseline Characteristics**			***p*-value**		
	phaco	phaco/MP-TSCPC	phaco/ECP	phaco vs phaco/ECP	phaco vs phaco/MP-TSCPC	phaco ECP vs phaco/MP-TSCPC
Number of eyes	19	20	25			
Mean Age (± SD)	66.75 ± 9.7	73.05 ± 14.9	72.68 ± 9.8	0.33	0.38	0.88
Age (Range)	52–83	38–91	49–93			
Sex (% Female)	63	55	48	0.32	0.60	0.64
IOP (mmHg ± SD)	14.30 ± 4.2	18.37 ± 4.6	15.78 ± 4.7	0.45	0.02	0.11
Medications (± SD)	1.75 ± 1.30	3.37 ± 1.30	2.84 ± 1.30	0.13	< 0.01^*^	0.20
Visual Acuity (± SD)	0.68 ± 0.54	0.84 ± 0.98	0.33 ± 0.23	0.20	0.80	0.57
Mean Follow-up Time (± SD) (days)	112.82 ± 7.87	256.39 ± 42.33	215.27 ± 34.36	0.01^*^	0.01^*^	0.67
**Type of Glaucoma, N (%)**						
Ocular hypertension	0 (0)	0 (0)	1 (4)	n/a	n/a	n/a
POAG	8 (42)	5 (25)	12 (48)	0.04^*^	0.25	1
PXG	2 (11)	0 (0)	2 (8)	0.82	n/a	n/a
Pigmentary	1 (5)	0 (0)	2 (8)	n/a	n/a	n/a
NTG	1 (5)	0 (0)	0 (0)	n/a	n/a	n/a
MMG	5 (26)	8 (40)	3 (12)	n/a	n/a	0.09
CACG	2 (11)	7 (35)	5 (20)	0.45	0.22	0.56
**Severity of Glaucoma, N (%)**						
Ocular hypertension	0 (0)	0 (0)	1 (4)	n/a	n/a	n/a
Mild	0 (0)	2 (11)	6 (24)	n/a	n/a	n/a
Moderate	4 (8)	6 (26)	10 (40)	0.32	0.45	0.53
Severe	15 (92)	12 (63)	8 (32)	< 0.01^*^	0.03^*^	0.28

*IOP* Intraocular pressure, *SD* Standard deviation, *mm Hg* millimeters of mercury, *N* Number of eyes, *POAG* Primary Open-Angle Glaucoma, *PXG* Pseudoexfoliation Glaucoma, *NTG* Normal Tension Glaucoma, *MMG* Mixed-mechanism glaucoma, *CACG* Chronic Angle-Closure Glaucoma

^*^ Indicates a significant difference

### Effectiveness

**Fig. 2:**
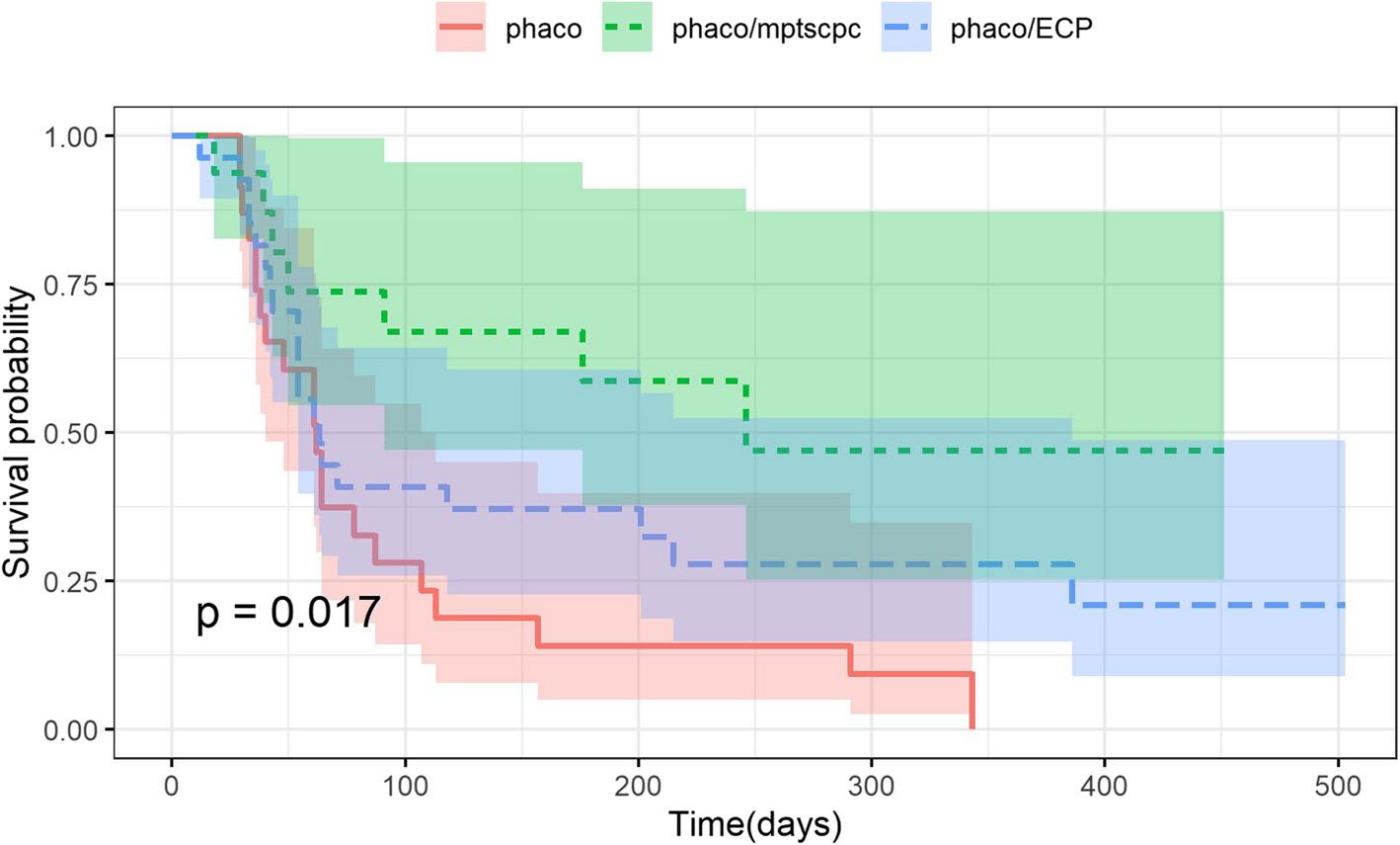
Kaplan–Meier curve comparing the cumulative probabilities of failure following phaco/ECP alone, phaco/MP-TSCPC, and phaco alone based on the IOP-reduction criteria. The shaded areas around each plot represent the 95% confidence bands. Failure was defined as reaching NLP vision at any point postoperatively, undergoing additional glaucoma surgery, or the inability to maintain ≥ 20% IOP reduction from baseline with IOP between 5–18 mmHg for two consecutive follow-up visits while maintaining ≤ baseline medications. The log-rank test was used to detect statistical differences between the three curves. IOP = intraocular pressure; phaco = phacoemulsification; MP-TSCPC = Micropulse Transscleral Cyclophotocoagulation (IRIDEX Corp., Mountainview, CA); ECP = Endoscopic Cyclophotocoagulation; mmHg = millimeters of mercury

A life table displaying the cumulative probability of survival in all three groups is displayed in Table 2. The cumulative probability of failure based on the IOP reduction was significantly lower in the phaco/MP-TSCPC group compared to the phaco alone group (*p* < 0.001, Fig. 2). Holding preoperative characteristics constant (age, sex, baseline IOP, number of medications, and type of glaucoma), the phaco alone group was 3.40 times as likely to reach failure at any time point compared to phaco/MP-TSCPC group (*p* = 0.005). Additionally, the cumulative probability of reaching failure at any time point was 1.98 times more likely following phaco/ECP compared to phaco/MP-TSCPC (*p* = 0.038). However, once the differences in preoperative IOP were accounted for through the Cox PH model, this difference only approached significance (*p* = 0.052). Patients who received phaco were 1**.**40 times more likely to reach failure as those who received phaco/ECP (*p* = 0.044, Table 3). IOP was found to be negatively correlated with the probability of failure in all Cox PH models (Table 3). Neither the age, sex, type of glaucoma, or number of preoperative medications of the patient was found to be correlated with the probability of failure in any of the Cox PH models (Supplementary Table 1). The cumulative probability of failure based on the goal IOP criteria was significantly higher in the phaco alone groups than in the phaco/MP-TSCPC (*p* < 0.001) or phaco/ECP (*p* = 0.024) groups. There was no significant difference in the cumulative probability of failure between the phaco/MP-TSCPC or phaco/ECP groups based on this set of criteria (*p* = 0.28) (Supplementary Fig. 1).

**Table 2:** Life table displaying the cumulative probabilities of survival in the phaco/ECP, phaco/MP-TSCPC, and phaco alone groups at 100 and 200 days postoperatively. Failure was defined as reaching NLP vision at any point postoperatively, undergoing additional glaucoma surgery, or the inability to maintain ≥ 20% IOP reduction from baseline with IOP between 5–18 mmHg for two consecutive follow-up visits while maintaining ≤ baseline medications

	**Survival Probability**	**95% CI**
**Phaco/ECP**		
100 ± 15 days	37.0%	(22.6%, 60.6%)
200 ± 15 days	32.4%	(18.6%, 56.6%)
**Phaco/MP-TSCPC**		
100 ± 15 days	67.0%	(47.0%, 95.5%)
200 ± 15 days	58.6%	(37.7%, 91.1%)
**Phaco**		
100 ± 15 days	18.8%	(5.4%, 65%)
200 ± 15 days	0	N/A

*CI* Confidence Interval, *phaco* phacoemulsification, *MP-TSCPC* Micropulse Transscleral Cyclophotocoagulation (IRIDEX Corp., Mountainview, CA), *ECP* Endoscopic cyclophotocoagulation

**Table 3:** Output of the pairwise log-rank tests and Cox PH Model

**Log Rank Test and Cox PH Model Outcomes**	**Hazard Ratio**	***p*-value**
**phaco vs phaco/MP-TSCPC**		
Log-Rank test		< 0.001*
Procedure	3.40	0.005*
Preoperative IOP	1.09	0.011*
**phaco vs phaco/ECP**		
Log-Rank test		0.037*
Procedure	1.40	0.044*
Preoperative IOP	1.11	0.004*
**phaco/ECP vs phaco/MP-TSCPC**		
Log-Rank test		0.038*
Procedure	1.98	0.052
Preoperative IOP	1.12	0.002*

Holding all else constant, the probability of achieving failure at any time point postoperatively with phaco alone was 3.40 times more likely than with phaco/MP-TSCPC (*p* = 0.005). Higher baseline IOP was found to be a statistically significant predictor of survival in comparing phaco/MP-TSCPC and phaco alone, with a one unit increase in baseline IOP reducing the hazard of clinical failure at any point in time by 9% (*p* = 0.022)

*phaco* phacoemulsification, *MP-TSCPC* Micropulse Transscleral Cyclophotocoagulation (IRIDEX Corp., Mountainview, CA), *ECP* Endoscopic cyclophotocoagulation, *PH* Proportional Hazards

^*^ Indicates a significant difference

The phaco/MP-TSCPC group achieved greater average IOP reduction than the phaco alone group at the month 3 (8.53 ± 6.8 vs 0.53 ± 1.65 mmHg, *p* < 0.01), and month 6 (6.80 ± 4.4 vs 1.86 ± 1.2, *p* = 0.02) visits. The phaco/MP-TSCPC group achieved greater average IOP reduction than the phaco/ECP group at week 6 (6.03 ± 6.8 vs 2.83 ± 9.3, *p* = 0.02) and month 3 (8.53 ± 6.8 vs 2.81 ± 4.0, *p* < 0.01). At 1 year, the mean IOP reduction was 1.0 ± 1.6 mmHg in the phaco alone group, 6.0 ± 4.3 mm Hg in the phaco/MP-TSCPC group, and 3.07 ± 5.3 in the phaco/ECP group (*p* = 0.12). While the phaco/ECP group achieved greater average IOP reduction than the phaco alone group from week 1 onwards, this difference did not achieve statistical significance (Table 4). The phaco/MP-TSCPC group achieved a significantly higher mean medication reduction than the phaco alone group at the 6-month visit (1.3 ± 0.8 vs -0.09 ± 0.83, *p* = 0.023, Table 5).

**Table 4:** Comparison of average IOP reductions between the phaco/ECP, phaco/MP-TSCPC, and phaco alone groups

**Mean IOP reduction (mm Hg ± SD)**				***p*-value**		
	phaco	phaco/MP-TSCPC	phaco/ECP	phaco vs phaco/ECP	phaco vs phaco/MP-TSCPC	phaco/ECP vs phaco/MP-TSCPC
Day 1 (% reduction)	-5.13 ± 9.2 (35.9)	0.53 ± 4.5 (2.9)	-3.13 ± 4.3 (-19.9)	0.69	0.16	0.39
*n*	*19*	*20*	*25*			
Week 1(% reduction)	0.04 ± 2.23 (0.3)	2.13 ± 7.3 (11.6)	4.68 ± 5.6 (29.7)	0.18	0.13	0.41
*n*	*17*	*20*	*24*			
Week 6 (% reduction)	0.72 ± 2.25(5.0)	6.03 ± 6.8 (32.8)	2.83 ± 9.3(17.9)	0.33	< 0.01*	0.02*
*n*	*19*	18	*19*			
Month 3 (% reduction)	0.53 ± 1.65 (3.7)	8.53 ± 6.8 (46.4)	2.81 ± 4.0 (17.8)	0.24	< 0.01*	< 0.01*
*n*	*16*	*18*	*21*			
Month 6 (% reduction)	1.86 ± 1.2 (1.3)	6.80 ± 4.4 (37.0)	2.70 ± 5.0 (17.1)	0.38	0.02*	0.08
*n*	*18*	*18*	*25*			
Year 1 (% reduction)	1.0 ± 1.6 (7.0)	6.0 ± 4.3 (32.7)	3.07 ± 5.3 (19.5)	0.24	0.10	0.10
*n*	*17*	*19*	*23*			

A negative IOP reduction indicates an increase in IOP postoperatively, *n* = number of patients at each follow-up visit

*IOP* Intraocular pressure, *mm Hg* millimeters of Mercury, *phaco* phacoemulsification, *MP-TSCPC* Micropulse Transscleral Cyclophotocoagulation (IRIDEX Corp., Mountainview, CA), *ECP* Endoscopic cyclophotocoagulation, *(SD)* Standard deviation

^*^ Indicates a significant difference

**Table 5:** Comparison of average medication reductions between the phaco/ECP, phaco/MP-TSCPC, and phaco alone groups

**Mean Med reduction (mean ± SD)**				***p*-value**		
	phaco	phaco/MP-TSCPC	phaco/ECP	phaco vs phaco/ECP	phaco vs phaco/MP-TSCPC	phaco/ECP vs phaco/MP-TSCPC
Day 1	0.27 ± .50	1 ± 0.87	0.36 ± 0.48	0.96	0.11	0.07
Week 1	0.58 ± 1.09	0.68 ± 0.70	0.79 ± 0.48	0.79	0.95	0.92
Week 6	0.13 ± 0.8	0.65 ± 0.53	0.82 ± 1.03	0.38	0.60	0.90
Month 3	-0.25 ± 1.5	0.47 ± 0.58	0.92 ± 0.47	0.22	0.54	0.72
Month 6	-0.09 ± 0.83	1.3 ± 0.8	1 ± 0.75	0.08	*0.03	0.82
Year 1	-0.2 ± 1.2	0.25 ± 1.1	0.62 ± 0.76	0.12	0.92	0.38

A negative IOP reduction indicates an increase in IOP postoperatively

*phaco* phacoemulsification, *MP-TSCPC* Micropulse Transscleral Cyclophotocoagulation (IRIDEX Corp., Mountainview, CA), *ECP* Endoscopic cyclophotocoagulation, *(SD)* Standard deviation

^*^ Indicates a significant difference

Changes in LogMAR visual acuity from baseline were not significantly different between the groups at any time point (Supplementary Table 2).

In terms of complications, 25% of the patients in the phaco/MPTSCPC group had anterior chamber inflammation at postoperative week 6, while 8% of patients in the phaco/ECP group and 0% of patients in the phaco alone group had inflammation at that time point (*p* = 0.31). All instances of inflammation were resolved by the 6-month follow-up visit. There were no instances of endophthalmitis, hypotony or retinal detachment. There were no instances of any long-term postoperative complications within the 1-year follow-up period (Table 6). Among the 64 eyes included in the study, there were 7 total cases of transient CME. The rates of CME did not differ significantly between groups at any follow-up visit, and all instances had resolved by the 6-month follow-up visit (Supplementary Table 3).

**Table 6:** Comparison of complication rates between the phaco alone, phaco/MP-TSCPC and phaco/ECP groups

**Complication Rates, N (%)**	phaco	phaco/MP-TSCPC	phaco/ECP	*p*-value (one-way ANOVA)
**Week 6**				
Inflammation	0 (0)	5 (25)	2 (8)	0.31
CME	0 (0)	0 (0)	0 (0)	n/a
Posterior Synechiae	0 (0)	0 (0)	0 (0)	n/a
Anterior Synechiae	0 (0)	0 (0)	0 (0)	n/a
Endophthalmitis	0 (0)	0 (0)	0 (0)	n/a
Hypotony	0 (0)	0 (0)	0 (0)	n/a
Retinal Detachment				
**Month 3**				
Inflammation	0 (0)	2 (10)	0 (0)	0.11
CME	0 (0)	0 (0)	0 (0)	n/a
Posterior Synechiae	0 (0)	0 (0)	0 (0)	n/a
Anterior Synechiae	0 (0)	0 (0)	0 (0)	n/a
Endophthalmitis	0 (0)	0 (0)	0 (0)	n/a
Hypotony	0 (0)	0 (0)	0 (0)	n/a
Retinal Detachment				
**Month 6**				
Inflammation	0 (0)	0 (0)	0 (0)	n/a
CME	0 (0)	0 (0)	0 (0)	n/a
Posterior Synechiae	0 (0)	0 (0)	0 (0)	n/a
Anterior Synechiae	0 (0)	0 (0)	0 (0)	n/a
Endophthalmitis	0 (0)	0 (0)	0 (0)	n/a
Hypotony	0 (0)	0 (0)	0 (0)	n/a
Retinal Detachment	0 (0)	0 (0)	0 (0)	n/a
**Year 1**				
Inflammation	0 (0)	0 (0)	0 (0)	n/a
CME	0 (0)	0 (0)	0 (0)	n/a
Posterior Synechiae	0 (0)	0 (0)	0 (0)	n/a
Anterior Synechiae	0 (0)	0 (0)	0 (0)	n/a
Endophthalmitis	0 (0)	0 (0)	0 (0)	n/a
Hypotony	0 (0)	0 (0)	0 (0)	n/a
Retinal Detachment	0 (0)	0 (0)	0 (0)	n/a

*N* Number of eyes, *phaco* phacoemulsification, *MP-TSCPC* Micropulse Transscleral Cyclophotocoagulation (IRIDEX Corp., Mountainview, CA), *ECP* Endoscopic cyclophotocoagulation, *CME* Cystoid Macular Edema

## Discussion

To the best of our knowledge, this is one of the first studies to examine the relative safety and effectiveness of MP-TSCPC and ECP as adjunct procedures combined with phacoemulsification. Prior studies have mostly demonstrated a limited IOP-lowering effect for phacoemulsification when performed alone. Arthur et al. demonstrated a mean IOP reduction of 2.5 mmHg from a baseline of 16.2 mmHg in a group of 37 patients with open-angle glaucoma [5]. Additionally, Poley et al. reported a mean IOP reduction of 2.7 mmHg at 1 year from a baseline of 17.8 mmHg in a group of 124 eyes. The authors noted that the magnitude of IOP reduction was highly correlated with the preoperative IOP [6].

The use of combined ECP and phacoemulsification has been well established in the literature [4, 7–11]. It has emerged as a preferred adjunct microinvasive procedure to phacoemulsification, primarily due to its convenience and low complication rate. Despite its popularity, some data on the long-term effectiveness of phaco/ECP as an IOP-lowering tool has been mixed. In a retrospective cohort study that consisted of 99 POAG patients, Perez et al. demonstrated that the surgical success rate after 1 year was significantly higher in the phaco/ECP group than in the phaco alone group (69.6% versus 40.0%, *p*= 0.004) [12]. Similarly, Francis et al. demonstrated mean IOP reductions of 2.7 mmHg and 0.9 mmHg in the phaco/ECP and phaco alone groups, respectively, at 3 years (*p*= 0.003). Interestingly, the group that underwent cataract extraction alone showed regression to the preoperative IOP after 2–3 years of follow up while the group that received phaco/ECP seemed to maintain the IOP reduction throughout the entire course of the study [13].

Other studies have raised into question the ability of phaco/ECP to consistently produce adequate IOP-reduction. In a retrospective study of over 300 eyes, Siegel et al. reported no significant difference in the IOP-lowering effect of phaco/ECP and phaco after 3 years of follow-up. Additionally, Lindfield et al. reported an average IOP reduction of 7.11 mmHg from a baseline of 21.54 mmHg at 18 months, with a mean medication reduction of 0 drops from a baseline of 1.98 drops [9]. While the IOP was significantly lowered at each follow-up visit, the authors noted that the reduction was not great enough for phaco/ECP to be considered an alternative for filtration surgery in high-risk eyes, rapidly progressive patients or when a low target pressure (< 14 mmHg) is indicated. With an IOP reduction of 3.07 mmHg in the phaco/ECP group, the reduction at 1 year in the present study was amongst the lowest reported in the literature, despite treating at least 300° of the ciliary processes in all cases. The preoperative medicated IOP here was 15.78 mmHg in the phaco/ECP group, which was amongst the lowest reported in the literature. The comparatively low mean IOP reductions observed here are likely attributable to the low preoperative IOPs, as preoperative IOP has consistently been demonstrated to correlate with the magnitude of IOP reduction.

The results of the present study suggest that phaco/MP-TSCPC provides at least equivalent IOP reduction to phaco/ECP without increasing the risk of postoperative complications. The KM survival curves along with the results of the Cox PH model seem to indicate a trend towards phaco/MP-TSCPC being more effective than phaco/ECP in terms of long-term IOP reduction. The Cox PH model indicates that phaco/ECP patients were 1.98 times more likely than the phaco/MP-TSCPC group to reach surgical failure at any point in time than the phaco/ECP group. Additionally, it demonstrates that other than preoperative IOP, no other pretreatment characteristics, such as type or stage of glaucoma, had a significant effect on the amount of IOP reduction observed. While there was a significant difference in the unadjusted survival curves of the patients who received phaco/MP-TSCPC and phaco/ECP (*p* = 0.038), this difference only approached significance when the differences in pretreatment characteristics were accounted for (*p* = 0.052). As with other studies comparing phaco/ECP and phaco, phaco/ECP appeared to provide comparatively greater long-term IOP control than phaco alone. While both the phaco/MP-TSCPC and the phaco/ECP groups outperformed the phaco alone groups in terms of survival rates, combining MP-TSCPC rather than ECP with phacoemulsification seemed to provide for even greater long-term IOP reduction. When the differences in preoperative characteristics were adjusted for, phaco/MP-TSCPC was 3.4 times less likely to reach surgical failure at any time point compared to phaco alone (*p* = 0.005). The long-term IOP-lowering effect in the phaco/ECP group seemed to be comparatively weaker, as performing phaco/ECP was only 1.4 times less likely than phaco alone to result in surgical failure at any time point (*p*= 0.038). Notably, the majority of patients included in the phaco/MP-TSCPC group in the present study had severe glaucoma (63%). The use of phaco/MP-TSCPC in this group provided a mean IOP reduction of 6.0 mmHg at 1 year postoperatively, with a mean IOP of 12.37 mmHg at the 1-year visit. Prior studies have demonstrated that the maintenance of IOP within this range is effective in reducing the advancement of visual field defects, particularly in patients with severe glaucoma [14]. The sustained IOP reduction observed in this group indicates that phaco/MP-TSCPC may potentially be a useful tool in attaining long-term IOP reduction in severe glaucoma patients. In the present study, the improvements in visual acuity and reductions in IOP seen in the phaco alone group were less pronounced than in prior studies. The mean preoperative IOP of 14.3 mmHg for the phaco alone group in the present study was amongst the lowest out of all the studies examined. This could potentially account for the seemingly smaller IOP-reducing effect observed, as preoperative IOP was correlated with amount of IOP reduction in the present study as well as in prior studies involving phacoemulsification [5, 6]. It is important to note that the PH model used in the present study indicated that both the phaco/MP-TSCPC (*p* = 0.005) and the phaco/ECP groups (*p* = 0.044) had comparatively greater IOP-lowering effect than the phaco alone group even when the differences in preoperative IOP were considered. The comparatively small improvement in visual acuity is possibly due to the large subset of severe glaucoma patients in this group. It is also possible that the relatively small sample size in this group was insufficiently powered to detect a significant improvement in visual acuity. In our cohort, post-operative complications were rare and were similar to those reported in the literature. Notably, all cases of postoperative inflammation resolved by the 6-month follow-up visit, and there were no new cases of CME in any group.

This study has several limitations. It is important to note that this study samples patients from a tertiary referral center, and we cannot exclude biased referral patterns resulting in sampling bias. There also exists a risk of selection bias, as the choice of which procedure to use was done at the discretion of each individual surgeon. Therefore, patients with uncontrolled IOPs were more likely to undergo a combined procedure while those with rather stable IOPs were more likely to undergo phaco alone. However, all of the phaco MP/CPC cases were done by a single surgeon, all of the phaco/ECP cases were done by another surgeon, and all of the phaco cases were performed by a collection of different surgeons.These surgeons do not routinely share patients or collaborate on cases. While the risk of selection bias certainly exists, it may be limited due to the makeup of surgeons performing the cases. Additionally, a Cox PH model was fitted in an attempt to account for differences in baseline characteristics potentially introduced by selection bias.

In addition, the study’s retrospective design, lack of data on patient adherence, sample size and follow-up duration may also limit generalizability. Additionally, although the overall heterogeneity in the baseline characteristics of the groups was partially accounted for using the Cox PH model, it still limits the overall generalizability of this study.

In conclusion, the results of the present study indicate that phaco/MP-TSCPC and phaco/ECP may be comparably safe and efficacious in reducing IOP when compared to phacoemulsification alone. Future studies on prospective randomized studies of combined MIGS procedures are needed to elucidate the optimal combination in different patient populations.

## Supplementary Information

**Additional file 1: Supplementary Table 1.** Non-Significant Hazard Ratios from Cox Proportional-Hazards Model.

**Additional file 2: Supplementary Figure 1.** Kaplan–Meier curve comparing the cumulative probabilities of failure following phaco/ECP alone, phaco/MP-TSCPC, and phaco alone based on the goal IOP criteria.

**Additional file 3: Supplementary Table 2.** Comparison of average acuity reductions between the phaco alone, phaco/MP-TSCPC and phaco/ ECP groups.

**Additional file 4: Supplementary Table 3.** Comparison of complication rates between the p﻿haco alone, phaco/MP-TSCPC and phaco/ECP groups.

## Data Availability

The datasets used and/or analysed during the current study are available from the corresponding author on reasonable request.

## References

[1] Dastiridou AI, Katsanos A, Denis P (2018). Cyclodestructive procedures in glaucoma: a review of current and emerging options. Adv Ther.

[2] Anand N, Klug E, Nirappel A, Solá-Del VD (2020). A review of cyclodestructive procedures for the treatment of glaucoma. Semin Ophthalmol.

[3] Fellman R, Mattox C, Ross KM, Vicchrilli S. Know the New Glaucoma Codes. EyeNet Mag. 2011:65–66. http://www.aao.org/eyenet/article/know-new-glaucoma-staging-codes?october-2011. Published online

[4] Dondelinger R (2013). Phacoemulsification systems. Biomed Instrum Technol.

[5] Arthur SN, Cantor LB, Wudunn D (2014). Efficacy, safety, and survival rates of IOP-lowering effect of phacoemulsification alone or combined with canaloplasty in glaucoma patients. J Glaucoma.

[6] Poley BJ, Lindstrom RL, Samuelson TW, Schulze R (2009). Intraocular pressure reduction after phacoemulsification with intraocular lens implantation in glaucomatous and nonglaucomatous eyes. Evaluation of a causal relationship between the natural lens and open-angle glaucoma. J Cataract Refract Surg.

[7] Sun W, Yu CY, Tong JP (2018). A review of combined phacoemulsification and endoscopic cyclophotocoagulation: efficacy and safety. Int J Ophthalmol.

[8] Siegel MJ, Boling WS, Faridi OS (2015). Combined endoscopic cyclophotocoagulation and phacoemulsification versus phacoemulsification alone in the treatment of mild to moderate glaucoma. Clin Exp Ophthalmol.

[9] Lindfield D, Ritchie RW, Griffiths MFP (2012). “Phaco-ECP”: Combined endoscopic cyclophotocoagulation and cataract surgery to augment medical control of glaucoma. BMJ Open.

[10] Waldman CW, Desai M, Rahman EZ, Eliassi-Rad B (2019). Combined endocyclophotocoagulation and phacoemulsification in patients with glaucoma of African descent. Med Hypothesis Discov Innov Ophthalmol.

[11] Uram M (1995). Combined phacoemulsification, endoscopic ciliary process photocoagulation, and intraocular lens implantation in glaucoma management. Ophthalmic Surg.

[12] Pérez Bartolomé F, Rodrigues IA, Goyal S (2018). Phacoemulsification plus endoscopic cyclophotocoagulation versus phacoemulsification alone in primary open-angle glaucoma. Eur J Ophthalmol.

[13] Francis BA, Berke SJ, Dustin L, Noecker R (2014). Endoscopic cyclophotocoagulation combined with phacoemulsification versus phacoemulsification alone in medically controlled glaucoma. J Cataract Refract Surg.

[14] The AGIS Investigators (2000). The Advanced Glaucoma Intervention Study (AGIS): 7. The relationship between control of intraocular pressure and visual field deterioration. Am J Ophthalmol.

