# Supplementary material for: Transscleral vs endoscopic cyclophotocoagulation: safety and efficacy when combined with phacoemulsification
Source: BMC Ophthalmol. 2023 Mar 30;23:129. doi: 10.1186/s12886-023-02877-6 (PMC10061713; doi:10.1186/s12886-023-02877-6)
Supplement: Supplementary file 1 — Additional file 1: Supplementary Table 1. Non-Significant Hazard Ratios from Cox Proportional-Hazards Model. [file 12886_2023_2877_MOESM1_ESM.pdf]

Supplementary Table 1: Non-Significant Hazard Ratios from Cox Proportional-Hazards Model

	Parameter	IOP Reduction ≥ 20% with IOP between 5 and 18 HR	*P* value
**Phaco vs phaco/MP-TSCPC**			
	Age	1.14	0.3
	Sex (Female)		0.5
	Male	0.88	
	Glaucoma Stage (Indeterminate)		0.3
	Mild	0.46	
	Moderate	0.24	
	Severe	0.06	
	Preoperative Medication burden	0.67	0.2
**Phaco vs phaco/ECP**			
	Age	0.924	0.4
	Sex (Female)		
	Male	0.960	
	Glaucoma Stage (Indeterminate)		0.2
	Mild	1.21	
	Moderate	0.98	
	Severe	0.45	
	Preoperative Medication burden	0.25	
**Phaco vs phaco/ECP**			
	Age	.96	0.4
	Sex (Female)		
	Male	1.11	
	Glaucoma Stage (Indeterminate)		0.4
	Mild	0.78	
	Moderate	1.11	
	Severe	1.19	
	Preoperative Medication burden	0.67	0.3
