# Supplementary figures and images for: Transscleral vs endoscopic cyclophotocoagulation: safety and efficacy when combined with phacoemulsification
Source: BMC Ophthalmol. 2023 Mar 30;23:129. doi: 10.1186/s12886-023-02877-6 (PMC10061713; doi:10.1186/s12886-023-02877-6)

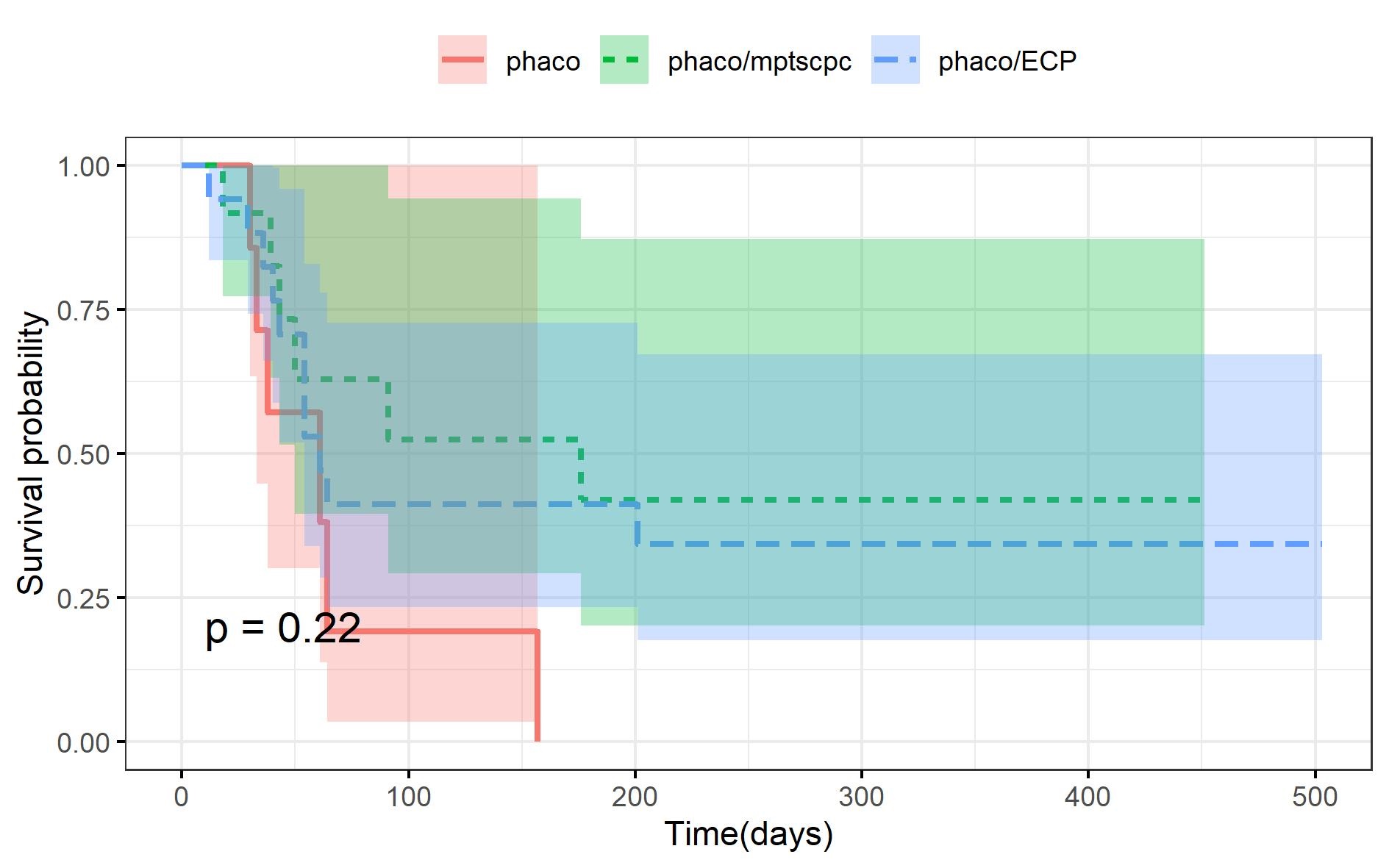

Supplement: Supplementary file 2 — Additional file 2: Supplementary Figure 1. Kaplan–Meier curve comparing the cumulative probabilities of failure following phaco/ECP alone, phaco/MP-TSCPC, and phaco alone based on the goal IOP criteria. [file 12886_2023_2877_MOESM2_ESM.jpg]
