# Supplementary material for: Transscleral vs endoscopic cyclophotocoagulation: safety and efficacy when combined with phacoemulsification
Source: BMC Ophthalmol. 2023 Mar 30;23:129. doi: 10.1186/s12886-023-02877-6 (PMC10061713; doi:10.1186/s12886-023-02877-6)
Supplement: Supplementary file 3 — Additional file 3: Supplementary Table 2. Comparison of average acuity reductions between the phaco alone, phaco/MP-TSCPC and phaco/ ECP groups. [file 12886_2023_2877_MOESM3_ESM.pdf]

Mean Acuity differences within the phaco, phaco/MP-TSCPC, and phaco/ECP groups

	Mean Acuity Difference (± SD)			p-value		
	phaco	phaco/MP-TSCPC	phaco/ECP	phaco vs phaco/ECP	phaco vs phaco/MP-TSCPC	phaco/ECP vs phaco/MP-TSCPC
Day 1	0.21 ± 0.35	0.20 ± 0.72	-0.07 ± 0.38	0.41	0.19	0.76
Week 1	0.34 ± 0.59	0.05 ± 0.63	0.12 ± 0.24	0.09	0.43	0.61
Week 6	0.58 ± 0.61	0.06 ± 0.40	0.02 ± 0.39	0.16	0.11	0.98
Month 3	0.44 ±0.45	0.13 ± 0.71	0.16 ± 0.30	0.59	0.53	0.99
Month 6	0.44 ± 0.32	0.15 ± 0.75	0.09 ± 0.25	0.25	0.45	0.95
Year 1	0.43 ± 0.33	0.49 ± 0.84	0.15 ± 0.27	0.60	0.98	0.45
Improvement from baseline	*p=0.30*	*p=0.22*	*p=0.35*			

Supplementary Table 2: Comparison of average acuity reductions between the phaco alone, phaco/MP-TSCPC and phaco/ ECP groups
phaco=phacoemulsification; MP-TSCPC=MP-TSCPC=Micropulse Transscleral Cyclophotocoagulation (IRIDEX Corp., Mountainview, CA); ECP=endoscopic cyclophotocoagulation; (SD)=standard deviation
