# Supplementary material for: Transscleral vs endoscopic cyclophotocoagulation: safety and efficacy when combined with phacoemulsification
Source: BMC Ophthalmol. 2023 Mar 30;23:129. doi: 10.1186/s12886-023-02877-6 (PMC10061713; doi:10.1186/s12886-023-02877-6)
Supplement: Supplementary file 4 — Additional file 4: Supplementary Table 3. Comparison of complication rates between the p﻿haco alone, phaco/MP-TSCPC and phaco/ECP groups. [file 12886_2023_2877_MOESM4_ESM.pdf]

**Complication Rates, N (%)**	phaco	phaco/MP-TSCPC	phaco/ECP	p-value (one-way ANOVA)
**Week 6**				
Inflammation	0 (0)	5 (25)	2 (8)	0.31
CME	2 (11)	2 (10)	3 (12)	0.53
Posterior Synechiae	0 (0)	0 (0)	0 (0)	n/a
Anterior Synechiae	0 (0)	0 (0)	0 (0)	n/a
Endophthalmitis	0 (0)	0 (0)	0 (0)	n/a
Hypotony	0 (0)	0 (0)	0 (0)	n/a
Retinal Detachment				
**Month 3**				
Inflammation	0 (0)	2 (10)	0 (0)	0.11
CME	0 (0)	1 (5)	1 (4)	n/a
Posterior Synechiae	0 (0)	0 (0)	0 (0)	n/a
Anterior Synechiae	0 (0)	0 (0)	0 (0)	n/a
Endophthalmitis	0 (0)	0 (0)	0 (0)	n/a
Hypotony	0 (0)	0 (0)	0 (0)	n/a
Retinal Detachment				
**Month 6**				
Inflammation	0 (0)	0 (0)	0 (0)	n/a
CME	0 (0)	0 (0)	0 (0)	n/a
Posterior Synechiae	0 (0)	0 (0)	0 (0)	n/a
Anterior Synechiae	0 (0)	0 (0)	0 (0)	n/a
Endophthalmitis	0 (0)	0 (0)	0 (0)	n/a
Hypotony	0 (0)	0 (0)	0 (0)	n/a
Retinal Detachment	0 (0)	0 (0)	0 (0)	n/a
**Year 1**				
Inflammation	0 (0)	0 (0)	0 (0)	n/a
CME	0 (0)	0 (0)	0 (0)	n/a
Posterior Synechiae	0 (0)	0 (0)	0 (0)	n/a
Anterior Synechiae	0 (0)	0 (0)	0 (0)	n/a
Endophthalmitis	0 (0)	0 (0)	0 (0)	n/a
Hypotony	0 (0)	0 (0)	0 (0)	n/a
Retinal Detachment	0 (0)	0 (0)	0 (0)	n/a
